# The Spatial Chemical Langevin Equation and Reaction Diffusion Master Equations: moments and qualitative solutions

**DOI:** 10.1186/s12976-015-0001-6

**Published:** 2015-02-27

**Authors:** Atiyo Ghosh, Andre Leier, Tatiana T Marquez-Lago

**Affiliations:** Integrative Systems Biology Unit Okinawa Institute of Science and Technology, Okinawa, Japan; Okinawa Institute of Science and Technology, Okinawa, Japan

**Keywords:** Reaction Diffusion Master Equation, Spatial Chemical Langevin Equation, Turing patterns, Noise-induced phenomena

## Abstract

**Background:**

It has been established that stochastic effects play an important role in spatio-temporal biochemical networks. A popular method of representing such stochastic systems is the Reaction Diffusion Master Equation (RDME). However, simulating sample paths from the RDME can be computationally expensive, particularly at large populations. Here we investigate an uncommon, but much faster alternative: the Spatial Chemical Langevin Equation (SCLE).

**Methods:**

We investigate moment equations and correlation functions analytically, then we compare sample paths and moments of the SCLE to the RDME and associated deterministic solutions. Sample paths are generated computationally by the Next Subvolume method (RDME) and the Euler-Maruyama method (SCLE), while a deterministic solution is obtained with an Euler method. We consider the Gray-Scott model, a well-known pattern generating system, and a predator–prey system with spatially inhomogeneous parameters as sample applications.

**Results:**

For linear reaction networks, it is well known that the first order moments of all three approaches match, that the RDME and SCLE match to the second moment, and that all approaches diverge at third order moments. For non-linear reaction networks, differential equations governing moments do not form a closed system, but a general moment equation can be compared term wise. All approaches match at the leading order, and the RDME and SCLE match at the second leading order. As expected, the SCLE captures many dynamics of the RDME where deterministic methods fail to represent them. However, areas of the parameter space in the Gray-Scott model exist where either the SCLE and RDME give qualitatively different predictions, or the RDME predicts patterns, while the SCLE does not.

**Conclusions:**

The SCLE provides a fast alternative to existing methods for simulation of spatial stochastic biochemical networks, capturing many aspects of dynamics represented by the RDME. This becomes very useful in search of quantitative parameters yielding desired qualitative solutions. However, there exist parameter sets where both the qualitative and quantitative behaviour of the SCLE can differ when compared to the RDME, so care should be taken in its use for applications demanding greater accuracy.

**Electronic supplementary material:**

The online version of this article (doi:10.1186/s12976-015-0001-6) contains supplementary material, which is available to authorized users.

## Background

Spatial-stochastic effects are increasingly found to play important roles throughout a range of biological scales, from intracellular and intercellular processes, to ecological and epidemiological scales [1-3]. A widely used approach to study stochastic spatial dynamics is the Reaction Diffusion Master Equation (RDME), in which space is partitioned discretely into a number of voxels. Diffusion can then occur between different voxels, and reactions can occur within voxels on the assumption that reactants are well-mixed.

The RDME is generally analytically intractable. However, there do exist some closed form solutions for systems involving monomolecular reactions [4]. Mean-field approaches provide some analytical tools to help understand systems with bimolecular reactions [5,6], but these do not provide exact solutions. It is possible to generate sample paths consistent with the RDME using a variety of spatial Stochastic Simulation Algorithms (SSSAs) [7-9]. While widely used, SSSAs suffer from several drawbacks. For example, there are spatial resolution limits under which artefacts in particle interactions might occur [10], and also some effects at boundaries might not be accurately captured [11]. However, a more significant drawback is the fact that SSSAs are event-driven algorithms. Thus, at large numbers of particles, the number of events per time step can become very large, and SSSAs become prohibitively slow. While one might argue that deterministic approaches might suffice in such regimes, it has been shown that stochastic effects can give rise to important effects here, such as noise-induced oscillations and patterns [3,12]. There exist alternative algorithms based on the RDME which are faster in such scenarios, but these sacrifice the exactness of the SSSA [13], and thus are not guaranteed to faithfully represent the behaviour of the RDME. In this paper, we investigate one such alternative: the Spatial Chemical Langevin Equation (SCLE). In the non-spatial setting, the Chemical Langevin Equation (CLE), can be derived from the Chemical Master Equation (CME) [14], which in turn can be derived from a microscopic description of chemical processes [15]. The CLE and CME can then be extended to the SCLE and RDME, respectively, by introducing diffusion analogously to linear reactions. The SCLE consists of a family of stochastic differential equations (SDEs), which have the advantage that they can be simulated with fixed time steps, thus shedding the computational overhead associated with the event-driven SSSAs. Furthermore, there are simple schemes available to simulate SDEs. However, very little work has been done on investigating the SCLE in detail (see [16,17]). Moreover, these studies do not incorporate diffusion terms in a manner consistent with linear reactions in the non-spatial case. Since diffusion might be viewed as a linear reaction, it is important to maintain a consistent formulation between diffusion and linear reactions when introducing diffusion into existing non-spatial models.

Differences between deterministic and Master Equation approaches have been well-studied (e.g. [18-20]). However, previous work on comparing Langevin and Master equations has concentrated on non-spatial settings using the CLE and the CME [21], and also in non-spatial systems with delay [22]. In particular, it has been found that for linear reaction networks, that the first two moments of CME and CLE match. It was also demonstrated numerically that the CME and CLE can give similar moments in a non-linear reaction with a population of the order of 100 [21], but no formal proof extrapolating to different scenarios or populations was provided. While the method of adding spatial interactions is a straightforward extension involving adding linear interactions to the non-spatial case, there remain some open questions as to the consequences of such an implementation. For example, in a spatial model, it is possible for populations of some species to be high in some voxels, and low in others. Thus, it remains uncertain as to whether inaccuracies arising from voxels with small populations will propagate through the system. Furthermore, there is the interesting question as to how spatial correlation functions might behave in the spatial setting, as there is no such analogue in the non-spatial case.

In this work, we address the applicability of the SCLE as a substitute for the RDME. Since a key aspect of spatial models includes spatial correlations of different species, we investigate moments and correlation functions of the RDME and SCLE, and compare these with deterministic solutions for general reaction networks. In general, the moment equations for systems with non-linear reactions do not form a closed set of equations, and cannot be solved without further assumptions on the underlying distribution of particle populations [23,24]. However, we can draw conclusions on the moments of the RDME and SCLE without closing this set of equations, namely by comparing each corresponding equation term-wise. We provide a thorough numerical investigation of the Gray-Scott model to investigate whether the SCLE is capable of capturing pattern formation driven by intrinsic noise.

## Results and discussion

### Formulation

We construct a space Ξ, partitioned into *N* disjoint voxels, *ξ*_1_, *ξ*_2_, …, *ξ*_*N*_. Consider a number, *S*, of distinct chemical species: *X*_1_, *X*_2_, …, *X*_*S*_. Each of these can react according to some reaction, of which there are *R* in total. We denote the population of *X*_*s*_ (*s* ∈ {1, …, *S*}) in each *ξ*_*n*_ ∈ Ξ by ${u}_n^{(s)}$. We can use these quantities to define the total population for each *ξ*_*n*_ ∈ Ξ as ${\mathbf{u}}_n={\left({u}_n^{(1)},{u}_n^{(2)},\dots, {u}_n^{(S)}\right)}^T$ (with *T* denoting the transpose of the vector) and also the total state of the system as an *S* x *N* matrix **U** = (**u**_1_, **u**_2_, …, **u**_*N*_). We allow the state of the system to change by reactions *r* ∈ {1, …, *R*}:

1 $$ {\displaystyle \sum_{s=1}^S{\alpha}_{sr}{X}_s\overset{a_r^{(n)}\left(\mathbf{U}\right)}{\to }}{\displaystyle \sum_{s=1}^S{\beta}_{sr}{X}_s} $$

where *α*_*sr*_ and *β*_*sr*_ are natural numbers defining the stoichiometries of the reaction *r*, and ${a}_r^{(n)}\left(\mathbf{U}\right)$ is the reaction propensity of reaction *r* occurring within voxel *ξ*_*n*_. Note that we set the propensity functions to be dependent on voxel attributes to allow for flexibility in our approach, e.g. to consider different sized voxels, or to introduce spatially-inhomogeneous reaction rates. Using the reactions defined in (1), we can define a stoichiometric matrix **V**^(*r*,*n*)^, with the same dimensionality as **U**, such that the occurrence of reaction *r* in voxel *ξ*_*n*_ causes a change of state from **U** to **U** + **V**^(*r*,*n*)^. We denote the value of **V**^(*r*,*n*)^ at the entry corresponding to species *s* in voxel *x* by ${v}_{s,x}^{\left(r,n\right)}$. Note that for typical systems, it will be the case that **V**^(*r*,*n*)^ is sparse, i.e. ${v}_{s,x}^{\left(r,n\right)}=0$ whenever *x* ≠ *n*, which can be for most values of *x*. However, it is helpful to define the stoichiometric matrix on a larger space since it allows for more compact expressions in our subsequent analysis. We allow species *s* to diffuse from *ξ*_*m*_ to *ξ*_*n*_ with propensity ${d}_{m,n}^{(s)}{u}_m^{(s)}$, where ${d}_{m,n}^{(s)}$ represents the microscopic diffusion rate of species *s* from voxel *m* to voxel *n*. The change of total state as a result of such a diffusion event is given by a matrix ${\mathbf{W}}_{m,n}^{(s)}$, which is of the same size as **U**_,_ has a value of 1 at the position corresponding to ${u}_n^{(s)}$ and −1 at the entry corresponding to ${u}_m^{(s)}$, and is zero everywhere else. We refer to the element of ${\mathbf{W}}_{m,n}^{(s)}$ corresponding to species *s’* in voxel *x’* by ${w}_{s\hbox{'},x\hbox{'}}^{\left(s,m,n\right)}$. In the proceeding analysis, for some collection of *n* (*n* ∈ *ℕ*) random variables, *X*_1_, *X*_2_, …, *X*_*n*_, we denote the expectation of ${\displaystyle \prod_{i=1}^n{X}_i}$ by $\left\langle {\displaystyle \prod_{i=1}^n{X}_i}\right\rangle$ and refer to this quantity as an *n*^th^ order moment. Furthermore, for random variables *X* and *Y*, we define the correlation of *X* and *Y* to be 〈*XY*〉, which can also be alternatively viewed as a second order moment. We continue our analysis by considering the RDME, then the SCLE and deterministic approaches. Immediately after each approach, we present the moment equation implied by the corresponding approach. For the reader’s convenience, definitions of the RDME, SCLE and deterministic approaches are found in equations (2), (4) and (6), respectively. The corresponding correlation functions are in (3), (5) and (7), respectively.

### Correlation functions from master equations

Using the definitions developed in the previous section, we can write out a corresponding RDME for the probability that the system is at some state **u** at a time *t*, i.e. *P*(**U** = **u**, *t*). We denote this by *P*(**u**, *t*) for brevity. This leaves us with the following form for the RDME:

2 $$ \begin{array}{c}\frac{\partial P\left(\mathbf{u},t\right)}{\partial t}={\displaystyle \sum_{r=1}^R{\displaystyle \sum_{n=1}^N\left({a}_r^{(n)}\left(\mathbf{u}-{\mathbf{V}}^{\left(r,n\right)}\right)P\left(\mathbf{u}-{\mathbf{V}}^{\left(r,n\right)},t\right)-{a}_r^{(n)}\left(\mathbf{u}\right)P\left(\mathbf{u},t\right)\right)}}\\ {}{\displaystyle \sum_{s=1}^S{\displaystyle \sum_{m=1}^N{\displaystyle \sum_{n=1}^N{d}_{m,n}^{(s)}\left({u}_m^{(s)}-{w}_{s,m}^{\left(s,m,n\right)}\right)P\left(\mathbf{u}-{\mathbf{W}}_{m,n}^{(s)},t\right)-{d}_{m,n}^{(s)}{u}_m^{(s)}P\left(\mathbf{u},t\right)}}}\end{array} $$

where the first and second lines represent contributions of reaction and diffusion events, respectively. To investigate various spatial phenomena, we consider the correlation function of the populations of species *x* in voxels *ξ*_*a*_ and *ξ*_*b*_, $\left\langle {u}_a^{(x)}{u}_b^{(x)}\right\rangle$. In situations with non-linear propensities, ODEs describing these correlation functions depend on higher order correlation functions, which in turn are related to correlation functions of ever-increasing order. Thus, we consider a general correlation function of order *O* represented by $\left\langle {\displaystyle \prod_{o=1}^O{u}_{n_o}^{\left({s}_o\right)}}\right\rangle$ where for each *o* we choose some *n*_*o*_ ∈ {1, …, *N*} and *s*_*o*_ ∈ {1, …, *S*}. We apply the operator ${\displaystyle \sum_{\mathbf{u}}{\displaystyle \prod_{o=1}^O{u}_{n_o}^{\left({s}_o\right)}}}$ to both sides of (2) to give the following ODE (see Additional file 1 for derivation):

3 $$ \begin{array}{c}\frac{d\left\langle {\displaystyle \prod_{o=1}^O{u}_{n_o}^{\left({s}_o\right)}}\right\rangle }{dt}={\displaystyle \sum_{r=1}^R{\displaystyle \sum_{n=1}^N\left\langle {a}_r^{(n)}\left(\mathbf{U}\right)\left({\displaystyle \prod_{o=1}^O\left({u}_{n_o}^{\left({s}_o\right)}+{v}_{s_o,{n}_o}^{\left(r,n\right)}\right)-{\displaystyle \prod_{o=1}^O{u}_{n_o}^{\left({s}_o\right)}}}\right)\right\rangle }}\\ {}+{\displaystyle \sum_{s=1}^S{\displaystyle \sum_{m=1}^N{\displaystyle \sum_{n=1}^N\left\langle {d}_{m,n}^{(s)}{u}_m^{(s)}\left({\displaystyle \prod_{o=1}^O\left({u}_{n_o}^{\left({s}_o\right)}+{w}_{s_o,{n}_o}^{\left(s,m,n\right)}\right)-{\displaystyle \prod_{o=1}^O{u}_{n_o}^{\left({s}_o\right)}}}\right)\right\rangle }}}\end{array} $$

where the expression is structured as in (2), and the first and second lines represent contributions from reaction and diffusion events, respectively. Observe that the leading order of both ${\displaystyle \prod_{o=1}^O\left({u}_{n_o}^{\left({s}_o\right)}+{v}_{s_o,{n}_o}^{\left(r,n\right)}\right)}$ and ${\displaystyle \prod_{o=1}^O\left({u}_{n_o}^{\left({s}_o\right)}+{w}_{s_o,{n}_o}^{\left(s,m,n\right)}\right)}$ is ${\displaystyle \prod_{o=1}^O{u}_{n_o}^{\left({s}_o\right)}}$. Consequently, the moments of the diffusion terms depend only on moments of order *O* and below. However, the order of the moments arising from reaction terms depends on the specific form of ${a}_r^{(n)}\left(\mathbf{U}\right)$. If all such reaction propensities are linear or constant, then (3) forms a closed set of equations. Otherwise, the moments of order *O* are dependent on moments of order *O + 1* or higher, and thus the set of ODEs in (3) is dependent on an infinite set of higher order ODEs, and cannot be solved without some extra assumptions on the process. For our purposes, we do not seek to solve (3), but only compare each ODE generated by (3) term-wise to corresponding ODEs obtained from the SCLE. This then provides some insight into how the spatial correlations compare between these approaches.

### Correlation functions from Langevin and deterministic equations

We consider the Langevin and deterministic regimes together in this section, since their analytical treatment is similar. Using the same established definitions from the case of the RDME, we can write out a set of SDEs for each chemical species in each voxel. Taken together, these provide a Langevin representation, which we take to be a counterpart to the RDME of the previous section. To arrive at such representations, one can either start from the Chemical Langevin Equation in the non-spatial setting (as in [21]) and introduce diffusion between voxels analogously to linear reactions. An alternative way to arrive at Langevin representations is to proceed directly by an expansion of the RDME [25]. Note that such representations assume that the population of various quantities are continuous variables, as opposed to the RDME approaches which preserve discreteness. The SCLE consists of the following coupled SDEs for the population of molecules of each species *s*_*o*_ in each voxel ${\xi}_{n_o}$:

4 $$ \begin{array}{c}d{u}_{n_o}^{\left({s}_o\right)}=\left({\displaystyle \sum_{r=1}^R{\displaystyle \sum_{n=1}^N\left({a}_r^{(n)}\left(\mathbf{U}\right){v}_{s_o,{n}_o}^{\left(r,n\right)}\right)+{\displaystyle \sum_{s=1}^S{\displaystyle \sum_{m=1}^N{\displaystyle \sum_{n=1}^N\left({d}_{m,n}^{(s)}{u}_m^{(s)}{w}_{s_o,{n}_o}^{\left(s,m,n\right)}\right)}}}}}\right)dt\\ {}+{\displaystyle \sum_{r=1}^R{\displaystyle \sum_{n=1}^N\left(\sqrt{a_r^{(n)}\left(\mathbf{U}\right)}{v}_{s_o,{n}_o}^{\left(r,n\right)}d{W}_r^{(n)}\right)+{\displaystyle \sum_{s=1}^S{\displaystyle \sum_{m=1}^N{\displaystyle \sum_{n=1}^N\left(\sqrt{d_{m,n}^{(s)}{u}_m^{(s)}}{w}_{s_o,{n}_o}^{\left(s,m,n\right)}d{W}_{mn}^{(s)}\right)}}}}}\end{array} $$

where each ${W}_r^{(n)}$ is a Wiener process used to represent noise in the occurrence of reaction *r* in voxel *ξ*_*n*_ . Similarly, each ${W}_{mn}^{(s)}$ incorporates the stochastic nature of diffusion of species *s* from voxel *m* to voxel *n*. We refer to the collection of SDEs represented by (4) as the SCLE.

An expression for an ODE describing $\left\langle {\displaystyle \prod_{o=1}^O{u}_{n_o}^{\left({s}_o\right)}}\right\rangle$ follows by applying Ito’s Lemma to (4) (see Additional file 1):

5 $$ \begin{array}{c}\frac{d\left\langle {\displaystyle \prod_{o=1}^O{u}_{n_o}^{\left({s}_o\right)}}\right\rangle }{dt}={\displaystyle \sum_{r=1}^R{\displaystyle \sum_{n=1}^N\left\langle {\displaystyle \sum_{o=1}^O\left({a}_r^{(n)}\left(\mathbf{U}\right){v}_{s_o,{n}_o}^{\left(r,n\right)}{\displaystyle \prod_{\begin{array}{l}o\hbox{'}=1\\ {}o\hbox{'}\ne o\end{array}}^O\left({u}_{n_{o\hbox{'}}}^{\left({s}_{o\hbox{'}}\right)}\right)}\right)}+\frac{1}{2}\left({\displaystyle \sum_{o=1}^O{\displaystyle \sum_{p=1}^O\left({a}_r^n\left(\mathbf{U}\right){v}_{s_o,{n}_o}^{\left(r,n\right)}{v}_{s_p,{n}_p}^{\left(r,n\right)}{\displaystyle \prod_{\begin{array}{l}o\hbox{'}=1\\ {}o\hbox{'}\ne o\\ {}o\hbox{'}\ne p\end{array}}^O\left({u}_{n_{o\hbox{'}}}^{\left({s}_{o\hbox{'}}\right)}\right)}\right)}}\right)\right\rangle }}\\ {}+{\displaystyle \sum_{s=1}^S{\displaystyle \sum_{m=1}^N{\displaystyle \sum_{n=1}^N\left\langle {\displaystyle \sum_{o=1}^O\left({d}_{m,n}^{(s)}{u}_m^{(s)}{w}_{s_o,{n}_o}^{\left(s,m,n\right)}{\displaystyle \prod_{\begin{array}{l}o\hbox{'}=1\\ {}o\hbox{'}\ne o\end{array}}^O\left({u}_{n_{o\hbox{'}}}^{\left({s}_{o\hbox{'}}\right)}\right)}\right)}+\frac{1}{2}\left({\displaystyle \sum_{o=1}^O{\displaystyle \sum_{p=1}^O\left({d}_{m,n}^{(s)}{u}_m^{(s)}{w}_{s_o,{n}_o}^{\left(s,m,n\right)}{w}_{s_p,{n}_p}^{\left(s,m,n\right)}{\displaystyle \prod_{\begin{array}{l}o\hbox{'}=1\\ {}o\hbox{'}\ne o\\ {}o\hbox{'}\ne p\end{array}}^O\left({u}_{n_{o\hbox{'}}}^{\left({s}_{o\hbox{'}}\right)}\right)}\right)}}\right)\right\rangle }}}\end{array} $$

where we have structured the expression in the same way as in (3), with the contribution from reaction and diffusion appearing in the first and second lines, respectively. The expression becomes clearer when seen in terms of binomial expansions of various terms in (3). The reaction terms in (5) are identical to the leading two orders of the reaction terms appearing in the expansion of (3). The same holds for the diffusion terms. Thus, each of the infinite set of ODEs representing correlation functions from the RDME and SCLE match up to the second leading order.

For the deterministic model, we consider the SCLE without any contribution from Wiener terms. That is, for each *s*_*o*_ in voxel ${\xi}_{n_o}$, we consider the following deterministic representation:

6 $$ d{u}_{n_o}^{\left({s}_o\right)}=\left({\displaystyle \sum_{r=1}^R{\displaystyle \sum_{n=1}^N\left({a}_r^{(n)}\left(\mathbf{U}\right){v}_{s_o,{n}_o}^{\left(r,n\right)}\right)+{\displaystyle \sum_{s=1}^S{\displaystyle \sum_{m=1}^N{\displaystyle \sum_{n=1}^N\left({d}_{m,n}^{(s)}{u}_m^{(s)}{w}_{s_o,{n}_o}^{\left(s,m,n\right)}\right)}}}}}\right)dt $$

The ODEs describing correlation functions and moment equations from (6) can be easily calculated, since in the deterministic scenario, expectation and products commute, i.e., $\left\langle {\displaystyle \prod_{o=1}^O{u}_{n_o}^{\left({s}_o\right)}}\right\rangle ={\displaystyle \prod_{o=1}^O\left\langle {u}_{n_o}^{\left({s}_o\right)}\right\rangle }$. However, to facilitate comparison with the RDME and SCLE, we consider ODEs of the same form as (3) and (5). The result follows directly from the derivation of (5) (see Additional file 1 for details):

7 $$ \begin{array}{c}\frac{d\left\langle {\displaystyle \prod_{o=1}^O{u}_{n_o}^{\left({s}_o\right)}}\right\rangle }{dt}={\displaystyle \sum_{r=1}^R{\displaystyle \sum_{n=1}^N\left\langle {\displaystyle \sum_{o=1}^O\left({a}_r^{(n)}\left(\mathbf{U}\right){v}_{s_o,{n}_o}^{\left(r,n\right)}{\displaystyle \prod_{\begin{array}{l}o\hbox{'}=1\\ {}o\hbox{'}\ne o\end{array}}^O\left({u}_{n_{o\hbox{'}}}^{\left({s}_{o\hbox{'}}\right)}\right)}\right)}\right\rangle }}\\ {}+{\displaystyle \sum_{s=1}^S{\displaystyle \sum_{m=1}^N{\displaystyle \sum_{n=1}^N\left\langle {\displaystyle \sum_{o=1}^O\left({d}_{m,n}^{(s)}{u}_m^{(s)}{w}_{s_o,{n}_o}^{\left(s,m,n\right)}{\displaystyle \prod_{\begin{array}{l}o\hbox{'}=1\\ {}o\hbox{'}\ne o\end{array}}^O\left({u}_{n_{o\hbox{'}}}^{\left({s}_{o\hbox{'}}\right)}\right)}\right)}\right\rangle }}}\end{array} $$

which lends itself to the same interpretation as (5). The deterministic representation only accounts for leading order terms of the correlation functions and moment equations.

### Implications of moment equations

From equations (3), (5) and (7), we can make some analytical and also some heuristic predictions on the behaviour of the RDME, SCLE and deterministic approaches. First note that all moment equations match at the leading order, and the RDME and SCLE match at the second leading order.

In the case of linear networks (i.e. ${a}_r^{(n)}\left(\mathbf{U}\right)$ is linear in **U** for all *r* and *n*), we have that all three approaches give rise to a set of moment equations, which are closed, and can thus be solved. Since all three approaches agree at the leading order, the mean population of all three approaches match. Similarly, since the RDME and SCLE match at second leading order, we can conclude that the variance of populations between the RDME and SCLE agree for linear networks, while the same cannot be said for deterministic approaches. Since all three approaches deviate at the third leading order, we anticipate that the three approaches will not agree at the level of third order moments and higher. These results have previously been considered in the non-spatial case [21], and their extension to the spatial case in this work is to be expected, since diffusion in space can be considered as a linear reaction in itself.

In the case that there are second order or higher terms of species populations appearing in ${a}_r^{(n)}\left(\mathbf{U}\right)$ for some *r* and *n*, then the equations (3), (5) and (7) do not form a closed set, since each equation of order *O* is dependent on orders of *O* + 1 or higher. Thus, in the system of (infinite) ODEs describing these moments, there will be terms where all three approaches disagree, since they match at most at the leading two orders. Thus, moments of populations with non-linear reaction propensities cannot be expected to match. These arguments also hold when applied to the spatial correlation functions within the system, since spatial correlation functions can be defined in terms of moments.

In the limit of large populations, each ODE is dominated by its leading order terms, and thus one can expect moments and correlation functions to match for systems with very large populations. At smaller populations we anticipate that the SCLE should provide a better approximation to the RDME than a comparable deterministic approach, since the RDME and SCLE match at two leading order terms, whereas a deterministic approach only matches at the leading order term. However it is as yet unclear at what numbers the SCLE and RDME might give similar moments. This problem is compounded by the fact that in spatial scenarios, it is possible for some areas of space to have large populations, with others having small populations, such as the case in Turing patterns. Thus, we proceed with a numerical investigation.

### The Gray-Scott model

On comparing the ODEs governing spatial correlation functions for the RDME, SCLE and deterministic approaches, it is clear that the same spatial correlations should not be expected from the three approaches, but it is uncertain when one approach should well-approximate the other. To provide an application with which we can illustrate the potential and limitations of each approach, we consider the Gray-Scott model [26], a widely used pattern generating system. While it was originally intended as a model of glycolysis [27], it has been shown that the model can generate several different patterns within a narrow parameter range [28], and that intrinsic noise can drive these patterns [12]. As such, it provides a good framework with which to probe differences between the three approaches.

For this we consider two reacting species, *U* and *V*. Denote the population of *U* and *V* in voxel *ξ*_*i*_ by *U*_*i*_ and *V*_*i*_, respectively. The Gray-Scott model is characterised by the following reactions occurring within each *ξ*_*i*_ ∈ Ξ:

8 $$ \begin{array}{l}U+2V\to 3V\\ {}V\to P\\ {}U\to Q\\ {}\varnothing \to U\end{array} $$

with reaction propensities within each voxel being *k*_1_*U*_*i*_*V*_*i*_(*V*_*i*_ − 1)/Ω^2^, *k*_2_*V*_*i*_, *k*_3_*U*_*i*_ and *k*_4_Ω, respectively. The Ω term serves as a parameter to vary the system size. We conduct numerical experiments in a 2D Cartesian square. Each side of the square consists of *L* (*L* ∈ ℕ) square voxels, each with length *h*, giving the system a total length of *Lh* per side. We refer to the diffusion constants of *U* and *V* as *D*_*U*_ and *D*_*V*_, respectively. Thus, the diffusion propensities of *U* and *V* from voxel *ξ*_*i*_ to neighbouring voxels are given by *D*_*U*_*U*_*i*_/*h*^2^ and *D*_*V*_*V*_*i*_/*h*^2^, respectively. In order to conduct numerical experiments, it is helpful to parameterise the reaction rates. Thus, we define the reaction rates in terms of two parameters *k* and *F*, and define *k*_1_ = 1, *k*_2_ = *F* + *k*, *k*_3_ = *F* and *k*_4_ = *F*. This parameterisation has been chosen so as to be consistent with previous studies of the Gray-Scott Model [12,28]. For all numerical investigations, we take Ω = 250, *D*_*U*_ = 2 × 10^− 5^, *D*_*V*_ = 1 × 10^− 5^, *h* = 0.01 and *L* = 50. For initial conditions, we use *U* = 250 and *V* = 0 everywhere, except in a centred box of 3×3 voxels, where we use *U* = 0 and *V* = 250. We operate in arbitrary length and time units in order to maintain consistency with previously published studies, which have followed this strategy. In order to simulate sample paths from the RDME, we implemented the next subvolume method [29]. To generate comparable paths from the SCLE and the deterministic approaches, we implemented the Euler-Maruyama method [30] and an Euler method, respectively. As expected, the SCLE simulations were executed much faster than corresponding RDME simulations. The typical computational time to simulate 2500 time units from the RDME was on the order of two days, whereas the corresponding time from the SCLE was on the order of half an hour (with a time step of 0.1 time units), as computed using MATLAB R2013a running on a 3.2 GHz Intel Xeon processor. Reducing the time step on the Euler-Maruyama and Euler methods to 0.025 time units was found to have no impact on the results.

**Figure 1:**
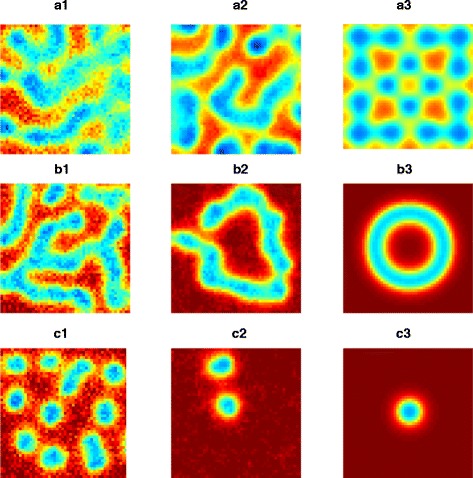
**Qualitatively different sample paths in the Gray Scott model depending on simulation method/numerical solution.** A selection of patterns generated by the Gray-Scott model from the RDME (panels numbered **1**), SCLE (panels numbered **2**) and deterministic equations (panels numbered **3**). Parameters used are as specified in the text, other than: a) *k* = 0.055, *F* = 0.025, b) *k* = 0.0625, *F* = 0.055 and c) *k* = 0.065, *F* = 0.04. All patterns are shown after a simulation time of 950 time units. Simulation time steps for the SCLE and deterministic approaches were 0.1 time units. The same results were found to hold with time steps reduced to 0.0025 time units.

In the area of parameter space where all three approaches generated patterns, typical patterns from the SCLE resembled something between the RDME and deterministic approaches (see Figure 1). That the qualitative patterns are not necessarily the same is not surprising, given one cannot expect the same correlation functions from all three approaches.

**Figure 2:**
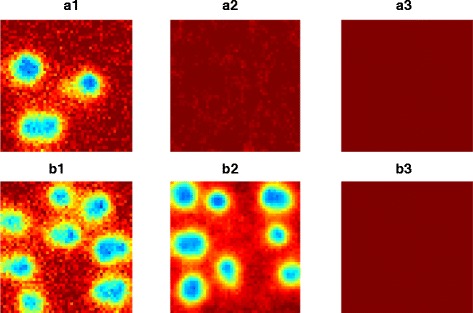
**The absence of patterns in the Gray Scott model depending on used simulation method/numerical solution.** Simulation output from the RDME (panels numbered **1**), SCLE (panels numbered **2**) and deterministic equations (panels numbered **3**) showing where the RDME predicts patterns but SCLE and deterministic approaches do not (subplots a), and cases where the RDME and SCLE predict patterns but the deterministic approach does not. Parameters are as specified in the text, with the addition of a) *k* = 0.06, *F* = 0.02 and b) *k* = 0.06, *F* = 0.025. Simulation time steps for the SCLE and deterministic approaches were 0.1 time units. The same results were found to hold with time steps reduced to 0.0025 time units.

**Figure 3:**
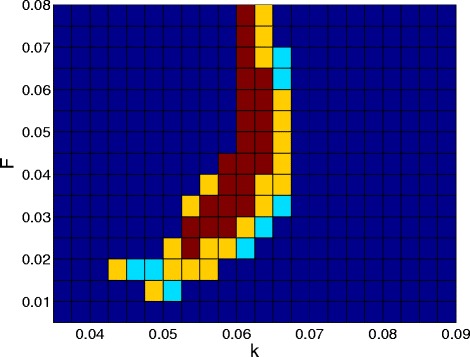
**Regions of parameter space where the RDME, SCLE and deterministic models predict patterns.** Regions where all three approaches generated patterns are given in red. Regions where only the RDME and SCLE generated patterns are given in yellow. Regions where only the RDME generated patterns are given in light blue. Dark blue symbolises that no patterns were found. Simulation time steps for the SCLE and deterministic approaches were 0.1 time units. The same results were found to hold with time steps reduced to 0.0025 time units. All other parameters are as indicated in the text.

However, it is not always the case that the three approaches predict the existence of patterns for the same parameters (see Figure 2). We observe three different scenarios: 1) All three approaches predict patterns, 2) Only the RDME and SCLE predict patterns and 3) Only the RDME predicts patterns. Such a result might be expected from the analytical expressions themselves. The region of parameter space where the RDME and the SCLE match is sizeable, and only in a few of the investigated parameters did the RDME predict patterns where the SCLE did not (see Figure 3). Our numerical results indicate that the RDME and SCLE give qualitatively similar results in the vast majority of the parameter space, however, there are cases where it can give qualitatively different results.

### A predator–prey system with a safe haven

To investigate first and second moments of a non-linear reaction system numerically, we consider a modified predator prey system in a space of 5 by 5 voxels, where prey are safe from predation in a small area. Such models can provide insight into the wider impact of implementing conservation schemes in local areas. We denote prey and predator species by *A* and *B*, respectively, and write their populations in voxel *i* as *A*_*i*_ and *B*_*i*_, respectively. The following reactions occur in every voxel:

9 $$ \begin{array}{l}A\to 2A\\ {}A+B\to 2B\\ {}A\to \varnothing \\ {}B\to \varnothing \end{array} $$

with corresponding propensities of *k*_1_*A*_*i*_, *k*_2_*A*_*i*_*B*_*i*_, *k*_3_*A*_*i*_ and *k*_4_*B*_*i*_, respectively. In voxel (1,1), we set *k*_*2*_ = 0, signifying that prey are safe from predators in this voxel. Furthermore, both *A* and *B* diffuse between voxels with the same diffusion rate *k*_*diff*_.

**Figure 4:**
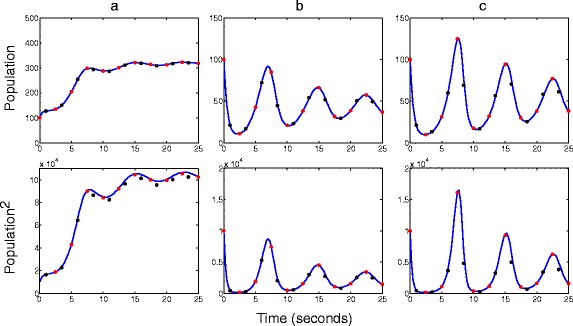
**A spatial predator prey system with a safe haven.** First moments (top row) and second moments (bottom row) of the predator–prey system described in (9). Voxels (1,1), (3,3) and (5,5) are shown in subplots **a**, **b** and **c**, respectively. Parameters used are *k*_*1*_ = 1[*A*]^−1^ *s* ^−1^, *k*_*2*_ = 0.02[*A*]^−1^[*B*]^−1^ *s* ^−1^ *k*_*3*_ = 0.25[*A*]^−1^ *s* ^−1^, *k*_*4*_ = 1[*B*]^−1^ *s* ^−1^ *k*_*diff*_ *=* 0.5[*A*]^−1^ *s* ^−1^ for *A* and *k*_*diff*_ *=* 0.5[*B*]^−1^ *s* ^−1^. Initial conditions are 100 individuals of *A* and *B* in every voxel. Time steps for the SCLE and deterministic approaches were 0.001 seconds. RDME results are shown as blue lines. SCLE and deterministic results are shown as red and black dots, respectively.

Sample paths from the RDME were generated by using the next subvolume method. Corresponding paths from the SCLE and deterministic approaches were generated by using the Euler-Maruyama and Euler methods, respectively. The population of *B* in voxels (1,1), (3,3) and (5,5) are shown in Figure 4, which shows first and second moments estimated from 10,000 realizations. The following parameters were used in the presented simulations: *k*_*1*_ = 1, *k*_*2*_ = 0.02, *k*_*3*_ = 0.25, *k*_*4*_ = 1, and *k*_*diff*_ *=* 0. Initial conditions of 100 individuals of *A* and 100 individuals of *B* were set in every voxel. At all times, values of the SCLE were found to interpolate those of the RDME and deterministic approaches. For example, at 10 seconds, the values of the second moment of *B* in voxel (1,1) from the RDME, SCLE and deterministic approaches was 85100, 84200 and 81800 rounded to three significant figures, respectively.

## Discussion

We conducted an investigation of the SCLE both analytically and numerically, with an emphasis on comparing moments, correlation functions and qualitative behaviour to the RDME and deterministic approaches. We show that for systems with linear reaction networks, the SCLE provides correct descriptions of first and second order moments, but not for third moments and higher. For non-linear cases, it cannot be guaranteed that the moments match.

In such non-linear scenarios, ODEs describing moments and correlation functions do not form a closed system of equations, but depend on higher order moment equations. Thus, these systems are not solvable without further assumptions being imposed on the system [23,24]. However, by comparing each equation term-wise, we showed that the RDME and SCLE match at the leading and second-leading orders, whereas deterministic approaches match only at the leading order. The implications of this depend on the specific reaction propensities in the network of interest. For linear networks, the RDME and SCLE match at the first and second moments, whereas deterministic approaches only represent first moments accurately. For non-linear networks, little can be said conclusively. These results are summarised in Table 1. Ascertaining the population size where this would lead to qualitative differences could not be performed analytically, so a numerical investigation was performed instead.

**Table 1:** **A term by term comparison of the SCLE and deterministic approaches with the RDME, and implications for the corresponding moments**

	**SCLE**	**Deterministic**
**Highest order matching moment (linear reactions)**	2nd Moment	1st Moment
**Highest order matching moment (higher order propensities)**	None	None
**Highest order matching term in moment ODEs (any propensity and moment)**	2nd Leading Order	Leading Order

The table summarises the terms matching between equations (3), (5) and (7).

Spatial studies of a predator–prey system showed that the SCLE can provide first and second order moments which closely match those of the RDME for populations on the order of 100 individuals (see Figure 4). These findings reinforce what was previously investigated in non-spatial settings [21]. The moments of the SCLE were found to interpolate between that of deterministic approaches and the RDME in all numerical investigations.

To demonstrate the applicability of the SCLE in capturing phenomena driven by intrinsic noise, we considered simulations of the Gray-Scott model [26,31]. While the qualitative solutions between the RDME and SCLE were typically similar, there were cases where they differed (see Figures 1 and 2). In particular, there were even some regions of parameter space where patterns might be observed in the RDME, but not in the SCLE (see Figures 2 and 3). Where the RDME, SCLE and deterministic approaches all predicted patterns, it was interesting to note that the resulting patterns obtained from the SCLE appeared to be an intermediate between patterns associated with the RDME and the PDE solutions.

Such results were conducted for populations of the order of a few hundred particles. In smaller populations, it is clear from equations (3), (5) and (7) that we cannot expect the moments nor correlation functions to match. It has recently been shown in the non-spatial case the CLE can be interpreted to give complex values for non-linear reactions at small populations [32], thus making it problematic to compare sample paths from master equations and Langevin equation at small populations, since it would involve comparing real and complex numbers with one another. In the spatial case, another artefact might appear in the SCLE: the fact that the SCLE admits continuous valued population sizes means that the notion of a particle being contained entirely within one voxel might be lost.

A key advantage the SCLE has over the RDME approach is computational efficiency. In executing the simulations in making Figures 1 and 3, the computational time required was on the order of days for simulations from the RDME, whereas analogous simulations were completed in the order of hours from the SCLE. The computational savings can be even more significant for larger systems, since SSSAs scale in computational speed according to a polynomial of the population size, whereas the speed of the Euler-Maruyama method is independent of the population size. As such, the main benefit of the SCLE is reached in the region where it gives the best accuracy, making it especially suitable for stochastic simulation on the macroscopic scales and parameter sweeps.

## Conclusions

We demonstrated that the SCLE captures many qualitative and quantitative characteristics of the RDME, which deterministic models fail to represent. The SCLE was found to be significantly faster to simulate than the RDME. Qualitative differences in behaviour of the RDME to the SCLE occurred for specific parameter settings, but such occurrences were uncommon. Analytical results demonstrate that the SCLE matches the RDME most at large population sizes. Thus, we anticipate that the SCLE should provide a strong framework for the simulation of reaction–diffusion systems with many particles.

## Additional file

Additional file 1:
**Derivations of correlation functions from the Reaction Diffusion Master Equation and the Spatial Chemical Langevin Equation.**

## Abbreviations

CLE: Chemical Langevin Equation
CME: Chemical Master Equation
RDME: Reaction Diffusion Master Equation
SCLE: Spatial Chemical Langevin Equation
