# Supplementary material for: The Spatial Chemical Langevin Equation and Reaction Diffusion Master Equations: moments and qualitative solutions
Source: Theor Biol Med Model. 2015 Feb 27;12:5. doi: 10.1186/s12976-015-0001-6 (PMC4351700; doi:10.1186/s12976-015-0001-6)
Supplement: Additional file 1: — Derivations of correlation functions from the Reaction Diffusion Master Equation and the Spatial Chemical Langevin Equation. [file 12976_2015_1_MOESM1_ESM.docx]

**Derivations of correlation functions from the Reaction Diffusion Master Equation and the Spatial Chemical Langevin Equation.**

**Derivation of (3)**

To obtain (3) apply $\sum_{\mathbf{u}} \prod_{o=1}^{O} u_{n_{o}}^{\left(s_{o}\right)}$ to both sides of (2). Evaluating the left hand side of (2) then leads to:

$$\begin{matrix} \sum_{\mathbf{u}} \prod_{o=1}^{O} u_{n_{o}}^{\left(s_{o}\right)}\frac{\partial P\left(\mathbf{u},t\right)}{\partial t}=\frac{\partial\sum_{\mathbf{u}} \prod_{o=1}^{O} u_{n_{o}}^{\left(s_{o}\right)}P\left(\mathbf{u},t\right)}{\partial t} \\ =\frac{d\left\langle\prod_{o=1}^{O} u_{n_{o}}^{\left(s_{o}\right)}\right\rangle}{dt} \end{matrix}$$

which matches the left hand side of (3). Now consider the right hand side of (2):

$\begin{matrix} \sum_{r=1}^{R} \sum_{n=1}^{N} \sum_{\mathbf{u}} \prod_{o=1}^{O} \left(u_{n_{o}}^{\left(s_{o}\right)}a_{r}^{\left(n\right)}\left(\mathbf{u}-\mathbf{V}^{\left(r,n\right)}\right)P\left(\mathbf{u}-\mathbf{V}^{\left(r,n\right)},t\right)-u_{n_{o}}^{\left(s_{o}\right)}a_{r}^{\left(n\right)}\left(\mathbf{u}\right)P\left(\mathbf{u},t\right)\right) \\ \sum_{s=1}^{S} \sum_{m=1}^{N} \sum_{n=1}^{N} \sum_{\mathbf{u}} \prod_{o=1}^{O} \left(u_{n_{o}}^{\left(s_{o}\right)}d_{m,n}^{\left(s\right)}\left(u_{m}^{\left(s\right)}-w_{s,m}^{\left(s,m,n\right)}\right)P\left(\mathbf{u}-\mathbf{W}_{m,n}^{\left(s\right)},t\right)-u_{n_{o}}^{\left(s_{o}\right)}d_{m,n}^{\left(s\right)}u_{m}^{\left(s\right)}P\left(\mathbf{u},t\right)\right) \end{matrix}$ .

We consider the reaction term only for an illustrative purpose. The diffusion term follows in the same way. First note that the Master Equation in (2) will prevent populations from becoming negative for physically relevant reaction propensities. Thus, we can extend the summation over **u** to all negative populations as well as positive populations without affecting the behaviour of the RDME. This then allows us to apply the transformation $\mathbf{u}\to\mathbf{u}+\mathbf{V}^{\left(r,n\right)}$, $\forall r\in\left\{1,...,R\right\}$ and $\forall n\in\left\{1,...,N\right\}$ to the first term of the first line without changing the evaluation of the sum in the first line. This leads to:

$\sum_{r=1}^{R} \sum_{n=1}^{N} \sum_{\mathbf{u}} \prod_{o=1}^{O} \left(\left(u_{n_{o}}^{\left(s_{o}\right)}+v_{s_{o},n_{o}}^{\left(r,n\right)}\right)a_{r}^{\left(n\right)}\left(\mathbf{u}\right)-u_{n_{o}}^{\left(s_{o}\right)}a_{r}^{\left(n\right)}\left(\mathbf{u}\right)\right)P\left(\mathbf{u},t\right)$ ,

which leads to the first line of (3) when the sum over **u** is executed. The diffusion term follows in the same manner.

**Derivation of (5) and (7)**

We use Ito's lemma as stated in [14] from the main text’s bibliography. Consider some number *n* of SDEs with *d* Wiener processes driving them. For $1\leq i\leq n$, put each SDE in the following form:

$$dY_{i}\left(t\right)=b_{i}\left(\mathbf{Y}\left(t\right)\right)dt+\sum_{j=1}^{d} \sigma_{ij}\left(\mathbf{Y}\left(t\right)\right)dW_{j}\left(t\right)$$

and define the matrix $c\left(\mathbf{Y}\left(t\right)\right)=\sigma\left(\mathbf{Y}\left(t\right)\right)\sigma\left(\mathbf{Y}\left(t\right)\right)^{T}$ . Then, for any twice continuously differentiable function , Ito’s lemma states:

$df\left(\mathbf{Y}\left(t\right)\right)=\left(\sum_{i=1}^{n} \frac{\partial f\left(\mathbf{Y}\left(t\right)\right)}{\partial x_{i}}b_{i}\left(\mathbf{Y}\left(t\right)\right)+\frac{1}{2}\sum_{i=1}^{n} \sum_{j-1}^{n} \frac{\partial^{2}f\left(\mathbf{Y}\left(t\right)\right)}{\partial x_{i}\partial x_{j}}c_{ij}\left(\mathbf{Y}\left(t\right)\right)\right)dt+M$ **,**

where *M* is a martingale whose specific form is unimportant to us, since we will take expectations of anyway. To obtain (5), use Ito’s Lemma with the SDEs from (4) with$f=\prod_{o=1}^{O} u_{n_{o}}^{\left(s_{o}\right)}$. This gives:

$d\prod_{o=1}^{O} u_{n_{o}}^{\left(s_{o}\right)}=\left(\begin{matrix} \sum_{o=1}^{O} \prod_{\begin{matrix} o'=1 \\ o'\neq o \end{matrix}}^{O} u_{n_{o'}}^{\left(s_{o'}\right)}\left(\sum_{r=1}^{R} \sum_{n=1}^{N} a_{r}^{\left(n\right)}\left(\mathbf{U}\right)v_{s_{o},n_{o}}^{\left(r,n\right)}+\sum_{s=1}^{S} \sum_{m=1}^{N} \sum_{n=1}^{N} \left(d_{m,n}^{\left(s\right)}u_{m}^{\left(s\right)}w_{s_{o},n_{o}}^{\left(s,m,n\right)}\right)\right) \\ +\frac{1}{2}\sum_{o=1}^{O} \sum_{p=1}^{O} \prod_{\begin{matrix} o'=1 \\ o'\neq o \\ o'\neq p \end{matrix}}^{O} u_{n_{o'}}^{\left(s_{o'}\right)}\left(\sum_{r=1}^{R} \sum_{n=1}^{N} a_{r}^{\left(n\right)}\left(\mathbf{U}\right)v_{s_{o},n_{o}}^{\left(r,n\right)}v_{s_{p},n_{p}}^{\left(r,n\right)}+\sum_{s=1}^{S} \sum_{m=1}^{N} \sum_{n=1}^{N} \left(d_{m,n}^{\left(s\right)}u_{m}^{\left(s\right)}w_{s_{o},n_{o}}^{\left(s,m,n\right)}w_{s_{p},n_{p}}^{\left(s,m,n\right)}\right)\right) \end{matrix}\right)dt+M$ **,**

where the first and second lines contain terms corresponding to the first and second order terms from , respectively. Taking expectations of , and rearranging terms gives rise to (5). To derive (7), either use the chain rule, or interpret the deterministic approach as one with the Wiener coefficients set to zero. In the latter case, equation (7) follows from by simply disregarding the second line, taking expectations, and rearranging.
